# Preventing the recurrence of depression with a Mediterranean diet supplemented with extra-virgin olive oil. The PREDI-DEP trial: study protocol

**DOI:** 10.1186/s12888-019-2036-4

**Published:** 2019-02-11

**Authors:** A. Sánchez-Villegas, B. Cabrera-Suárez, P. Molero, A. González-Pinto, C. Chiclana-Actis, C. Cabrera, F. Lahortiga-Ramos, M. Florido-Rodríguez, P. Vega-Pérez, R. Vega-Pérez, J. Pla, M. J. Calviño-Cabada, F. Ortuño, S. Navarro, Y. Almeida, J. L. Hernández-Fleta

**Affiliations:** 1 Nutrition Research Group, Research Institute of Biomedical and Health Sciences, University of Las Palmas de Gran Canaria, Paseo Blas Cabrera Felipe Físico s/n, 35016 Las Palmas de Gran Canaria, Spain; 2 Biomedical Research Center Network on Obesity and Nutrition (CIBERobn) Physiopathology of Obesity and Nutrition, Institute of Health Carlos III, Madrid, Spain; 3 Psychiatry and Clinical Psychology Service, Hospital General de Gran Canaria Dr. Negrin, Las Palmas de Gran Canaria, Spain; 4 Department of Psychiatry and Medical Psychology, University Clinic of Navarra, Pamplona, Spain; 5 IDISNA (Instituto de Investigación Sanitaria de Navarra), Pamplona, Spain; 6 Psychiatry and Clinical Psychology Service, Hospital Universitario de Alava, Vitoria, Spain; 7 Biomedical Research Center Network on Mental Health (CIBERsam), Institute of Health Carlos III, Madrid, Spain; 8 Consulta Dr. Chiclana, Madrid, Spain

**Keywords:** Mediterranean diet, Extra-virgin olive oil, Recurrence of depression, Clinical trial

## Abstract

**Background:**

The role of dietary patterns in the prevention of unipolar depression has been analyzed in several epidemiological studies. The primary aims of this study are to determine the effectiveness of an extra-olive oil-enriched Mediterranean diet in reducing the recurrence of depression and improving the symptoms of this condition.

**Methods:**

Multicenter, two-arm, parallel-group clinical trial. Arm 1, extra-virgin olive oil Mediterranean diet; Arm 2, control group without nutritional intervention. Dieticians are in charge of the nutritional intervention and regular contact with the participants. Contacts are made through our web platform (https://predidep.es/participantes/) or by phone. Recurrence of depression is assessed by psychiatrists and clinical psychologists through clinical evaluations (semi-structured clinical interviews: Spanish SCID-I). Depressive symptoms are assessed with the Beck Depression Inventory. Information on quality of life, level of physical activity, dietary habits, and blood, urine and stool samples are collected after the subject has agreed to participate in the study and once a year.

**Discussion:**

To the best of our knowledge, the PREDI-DEP trial is the first ongoing randomized clinical trial designed to assess the role of the Mediterranean diet in the prevention of recurrent depression. It could be a cost-effective approach to avoid recurrence and improve the quality of life of these patients.

**Trial registration:**

The study has been prospectively registered in the U.S. National Library of Medicine (https://clinicaltrials.gov) with NCT number: NCT03081065.

**Electronic supplementary material:**

The online version of this article (10.1186/s12888-019-2036-4) contains supplementary material, which is available to authorized users.

## Background

Unipolar depression is one of the leading global causes of disability-adjusted life years (DALYs) [1] and, in 2016, one of the leading causes of Years Lived with Disability (YLD) [2].

Prevention of depression recurrence is an essential goal in the management of depressive patients. Pharmacological treatment strategies are common to help prevent the risk of recurrence, as well as other alternatives, e.g., psychological interventions, which have shown promising outcomes [3].

Besides pharmacological and psychotherapeutic approaches, other interventions based on lifestyle changes, e.g., diet, physical activity, or alcohol and drug limitations, can be helpful can be helpful as part of the treatment of these patients. A body of research suggests the beneficial role of these factors in the etiopathogenesis of the condition and their potential utility for its management [4, 5].

Over the last years, several epidemiological studies have analyzed the role of dietary patterns, foods, food groups and nutrients, as factors that can help prevent unipolar depression. A recent systematic review and dose-response meta-analysis of prospective studies shows that higher quality diets associate with lower risks of developing depressive symptoms, although the authors believe that this hypothesis has to be further tested with prospective studies and randomized controlled trials [6]. Being an emerging promising field of research within nutritional epidemiology, to date there is still scarce evidence [7–9]. One of the dietary factors that has been inversely associated with depression is the adherence to the Mediterranean Dietary Pattern (MDP). Several cohort studies report an inverse relationship between following this healthy dietary pattern and the risk of developing depression [10–12]. Furthermore, two clinical trials carried out with depressive patients found significant improvement regarding depressive symptoms in patients assigned to the MDP [13, 14].

To our knowledge there are not clinical trials specifically designed to assess the role of a nutritional intervention based on the Mediterranean diet in the prevention of depression recurrence.

### Objectives

The primary objective of this study, the PREDI-DEP trial, is to investigate the effectiveness of following an extra-virgin olive oil-enriched Mediterranean diet with the risk of depression recurrence and improvement of residual depressive symptoms in participants with previous episodes of mayor depression. The secondary objectives are to analyze the effect of the Mediterranean nutritional intervention in the quality of life, various biochemical parameters and changes on the microbiota of the participants. We also aim to test the relationship between the nutritional intervention with an extra-virgin olive oil Mediterranean diet with the reduction in the risk of medical and psychiatric comorbidities in patients with a previous diagnosis of unipolar depressive disorder.

## Materials and methods

### Design

Multicenter, two-arm, parallel-group clinical trial. One group of patients (arm 1) is assigned to a Mediterranean diet supplemented with extra-virgin olive oil and the second group (control) (arm 2) has no nutritional intervention. The participants are recruited from four centers across Spain: Hospital Dr. Negrín (Las Palmas de Gran Canaria), Clínica Universidad de Navarra (Pamplona), Hospital Universitario (Vitoria), and Clínica Dr. Chiclana (Madrid). The intervention period lasted two years.

### Participant eligibility

#### Inclusion criteria

The inclusion criteria were established after an exhaustive review of the scientific literature and consensus among all participating psychiatric and clinical psychologists. Study subjects are aged ≥18 years, has had a previous major depressive episode within the last five years and are in a stage of total or partial remission within the last six months, based on DSM-5 criteria. One participant who has undergone a single depression episode is included for every three participants who had undergone two or more episodes.

#### Exclusion criteria

Table 1 shows the exclusion criteria in the PREDI-DEP trial:

**Table 1:** Exclusion criteria in the PREDI-DEP trial

1. Presence of comorbid psychiatric disorders (current mania/hypomania or a history of bipolar disorder, psychosis or schizophrenia, predominant anxiety disorder, primary personality disorder, substance abuse or eating disorder)	
2. Suspicion of depression (Score > 18 in Beck Depression Inventory) [16]	
3. Participants with severe medical conditions with low survival	
4. Participants with history of food allergy with hypersensitivity to any of the components of olive oil or nuts, presence of disorders of chewing or swallowing or a low predicted likelihood of changing dietary habits according to the Prochaska and DiClemente stages of change model [35]	
5. Institutionalized patients and those participants who lack autonomy	

### Patient recruitment

In a first phase of the selection process, we extract names of potential participants from the clinical records of the hospitals or health centers who are willing to collaborate with the study. Next, the participating psychologists and psychiatrists review the clinical records individually to identify the subjects that comply with the inclusion criteria. The potential participants are contacted by phone or during a clinical visit. When a candidate agrees to participate, a face-to-face interview with the specialist is carried out to exclude participants who do not meet the criteria. Besides the semi-structured clinical interview (Spanish SCID-I) [15], participants also self-complete the Beck Depression Inventory to assess depressive symptoms [16]. Information about the participant is completed with questions from the eligibility questionnaire, such as medical conditions or problems related with the adherence to the Mediterranean diet. During the first visit, participants receive a brief explanation of the study, are informed that are going to be given extra-virgin olive oil for the duration of the trial at no cost, and a signed informed consent is obtained. All study participants are asked to provide blood samples (previous appointment with the hospital) and a subgroup of participants is instructed to also provide urine samples (recruitment centers: Vitoria, Pamplona) and stool samples (recruitment center: Vitoria).

### Randomization

Study participants are randomly assigned to one of two groups (Mediterranean diet or control) once their data are included in a centralized computer system by the specialists. Various stratification factors are considered for the randomization, sex, age group (< 65 years or ≥ 65 years) and recruitment center. At baseline, psychiatrists and clinical psychologists are blinded to the allocation of the participants, following the CONSORT guidelines for randomized trials to prevent selection biases.

### Intervention with the Mediterranean diet and control group

Full-time registered dietitians with experience in the PREDIMED trial are responsible for the dietary intervention in the PREDI-DEP study. The PREDIMED trial is a landmark trial of intervention with a Mediterranean diet in participants at high risk of cardiovascular disease [17, 18]. The methodology in PREDI-DEP is similar to that in PREDIMED.

Participants allocated to the Mediterranean diet receive intensive training on Mediterranean diet and supplemental foods (Table 2), as well as extra-virgin olive oil (one liter of extra-virgin olive oil rich in polyphenols every two weeks), at no cost. The authors have no conflict of interest with any food company.

**Table 2:** Intervention with Mediterranean diet

Intervention	Periodicity	Instrument
Free food supply	Quarterly	Collected by participants in medical centers
Book and information regarding Mediterranean Diet	Initial	Collected by participants in medical centers
Buying foods list, plan of meals, cooking recipes, menus	Quarterly	Collected by participants in medical centers
Compliance degree, personalized goal achivement	Quarterly	Telephone
Mediterranean Diet news	Monthly	Web/e-mail
Clarification of doubts, suggestions	Any moment	Any instrument

At the beginning of the trial, the dieticians thoroughly explain the reasons to follow a Mediterranean diet to each participant and negotiate changes in his/her diet, working with the subject to determine what he or she considers an attainable goal. The dietician does this every three months by calling the participant over the phone. To assess adherence to the Mediterranean diet and enhance future adherence (Additional file 1), the dietitians use a validated 14-point Mediterranean Diet Assessment Screener (MEDAS) [19], which was employed in the PREDIMED trial.

Every three months the participants receive written material with information on key Mediterranean foods and seasonal shopping lists, menus and specific recipes for a typical week. This material is discussed in detail with the dietitians.

The website developed for this study (https://predidep.es/participantes/) is updated monthly with news related to the Mediterranean diet and its health effects. The dieticians take into account any doubt or suggestion made by the participants at any time of the intervention period.

No nutritional intervention is employed with the control group. These participants have an email and access to the website of the project, but with limitations on the contents related with the intervention. To prevent these subjects from withdrawing from a study an incentive at trial termination is offered.

### Data collection and measurements

Table 3 shows the variables, their corresponding time points and the researchers implicated in the collection of the data.

**Table 3:**
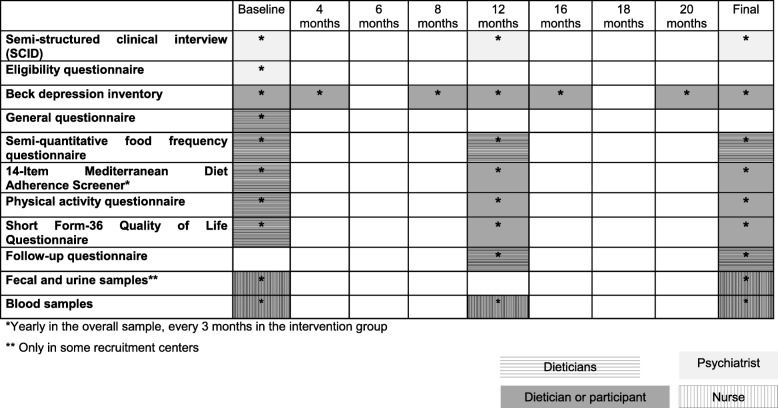
Exposures and outcomes measures, timepoints and researchers implicated in the collection of data

The dieticians are in charge of the nutritional interventions (Mediterranean diet), as well as for the regular contact and follow-up of the participants. Contacts are made using current technologies such as our web platform (https://predidep.es/participantes/), phone calls, or email. When a participant is not familiarized with these technologies, the dieticians use postal mail to send all the information.

### Assessment of exposure

#### Baseline information

The dieticians have the first contact with the participants over the phone to inform them in which arm they have been allocated and give instructions to the subjects who have been assigned to the Mediterranean diet as where and when to collect the food supply. Moreover, the dieticians complete the information for each participant through: 1) a general questionnaire; 2) a physical activity questionnaire; 3) a semi-quantitative food frequency questionnaire (FFQ); 3) the 14-Item Mediterranean Diet Adherence Screener (MEDAS), and 4) the Short Form-36 Quality of Life Questionnaire (SF-36).

#### Dietary assessment

Diet is assessed with a semi-quantitative FFQ validated in Spain [20]. The questionnaire covers 137 foods and is completed at baseline and yearly by the dietitians over the phone. The estimation of nutrient intake will be calculated as frequency multiplied by nutrient composition of the specified portion for each food item, using an ad hoc computer program developed specifically for this purpose. The repeated collection of dietary data allows us to use the PREDI-DEP trial as a unique setting for subsequent cohort studies, analyzed as a prospective observational follow-up study with repeated measurements of diet, thus improving the quality of our dietary assessment.

Changes in adherence to the Mediterranean diet is assessed through the MEDAS questionnaire at baseline and yearly in the control group and every three months in the Mediterranean diet group.

#### Assessment of the physical activity

Physical activity is assessed using a validated physical activity questionnaire with seventeen activities [21]. Leisure activities will be computed by assigning an equivalent metabolic score to each activity, multiplied by the time spent in each one and adding up all activities. The baseline questionnaire is completed over the phone. Yearly, participants complete the same questionnaire using the website of the project or by phone with the help of the dietician if they request it.

#### Assessment of other variables

Information regarding socioeconomic (educational level, employment and marital status), anthropometric (weight, height, and waist and hip circumferences), life-style (tobacco or history of illegal drug use), and medical characteristics of the participants, including medication, family history of mental disorders or the use of psychotherapy or relaxation techniques are obtained from the general questionnaire at baseline. This information is updated on a yearly basis using follow-up questionnaires.

### Outcome assessment of the nutritional intervention

#### Recurrence of depression

The clinical evaluations by specialists are limited to yearly follow-up visits that consist of the same examinations performed at baseline. If any suspicion of depression is detected through the Beck depression inventory or the subject him/herself communicates it, the specialist arranges an appointment with the participant. Specialists review the clinical records of participants lost to follow-up in order to assess possible cases of recurrence of depression not reported by the participants or detected by their specialists at follow-up.

#### Assessment of depressive symptoms

Depressive symptoms are assessed using the Beck Depression Inventory validated in Spain, which includes 21 questions with four possible answers sorted according to symptom severity [16].

Every four months, the participants complete this questionnaire over the phone with the aid of the dietitians or by themselves through the website. Exceptionally, at the beginning of the study or once a year the inventory is completed in the psychiatrist’s consulting room.

#### Quality of life assessment

Quality of life is assessed at baseline and yearly (over the phone or through the website) with the Spanish version of the SF-36 [22], a general, widely used, and thoroughly validated health scale. It contains 36 items that measure eight multi-item domains of health status: 1) physical functioning, 2) role limitations due to physical health problems (role-physical), 3) bodily pain, 4) general health perceptions, 5) vitality, 6) social functioning, 7) role limitations due to emotional problems (role-emotional), and 8) mental health. Domains 1 to 4 of the questionnaire deal with physical aspects, while domains 5 to 8 measure psychological features. For each parameter, scores will be coded, added up and transformed into a scale from 0 (the worst possible condition) to 100 (the best possible condition). For bodily pain domain a score of 100 implied complete tolerance or absence of pain.

### Biological samples

Blood, urine and stool samples are collected at baseline and yearly during medical visits. EDTA plasma tubes, buffy coat, and serum are collected and aliquots are kept at − 80 °C. All the samples are correctly identified and labeled with an alphanumeric code.

Biological compliance markers (plasma proportions of oleic and α-linolenic acid and urinary levels of tyrosol, hydroxytyrosol, resveratrol and ethanol) will be measured randomly in participants from the two arms of the trial at baseline and at the end of the study.

### Sample size and data analyses

The original sample size calculation indicated that 250 people per group were required (assuming an attrition of 5%) to provide a statistical power of 80% for detecting a relative risk reduction of 30% in the Mediterranean diet group versus the control group for a two-year follow-up period. The assumed recurrence rate was 50% for the control group and 35% for the group assigned to the Mediterranean diet. The relative risk reduction of 30% was slightly lower than the observed in the SUN cohort study. In this latter study, we found a 40% risk reduction of depression comparing extreme quintiles of adherence to the Mediterranean Diet Score [23] and the observed among diabetic participants of the PREDIMED trial after three years of intervention with the Mediterranean diet supplemented with nuts [24].

A researcher blinded to the conditions of the intervention will carry out the analysis of the data. Intention-to-treat analysis will be done. For each participant we will compute person-years of follow-up from the study inclusion date to the date of recurrence of the depression or study termination, whichever comes first.

Log Rank analysis will be used to assess the effect of the intervention on the risk of depression recurrence. If distribution differences of baseline characteristics are detected, the Cox proportional-hazards regression models will be used to check by recruitment center, age group and sex, and adjusting for possible confounding factors (educational level, marital status, prevalence of diseases, body mass index, tobacco use, leisure-time physical activity, alcohol intake, total energy intake, type, dose and use of antidepressants, psychotherapy, presence, number, and time since last of depressive symptoms/episodes, and family history of mental disorders). Hazard ratios (HR) and 95% confidence intervals (CI) will be calculated considering the control group as reference.

We will also carry out a protocol analysis [25]. Categories of adherence to the Mediterranean diet will be defined using information from the FFQ and MEDAS, and Cox regression models will be fitted. Marginal structural models (inverse probability of exposure-weighted estimators) will be fitted to exposure measures that vary over time [26]. Finally, to examine quantitative variables such adherence to the Mediterranean diet scorings [27], other analysis will be explored, e.g., smoothing splines and regression analysis based on fractional polynomials [28, 29].

Changes in residual depressive symptoms, quality of life or biochemical parameters were evaluated through Generalized Estimating Equation (GEE) models and will be adjusted for possible confounding factors and baseline values of depressive symptoms, quality of life or each biochemical parameter, respectively.

Subgroup and sensitivity analyses will be done under different assumptions. To assess a possible interaction between the intervention and some variables (e.g., age, sex or the presence of non-communicable diseases), product terms will be introduced in the multivariable models. *P* values for the interaction will be calculated using the log-likelihood ratio test.

Multiple imputation will be employed for handling missing data [30, 31].

### Monitoring of Data

Three independent clinical trial experts in Nutrition, Psychiatry and Statistics made up the data monitoring committee (DMC). They will hold an annual meeting to review the implementation of the protocol, monitor trial progress, and recommend the continuation or termination of the study based on safety, outstanding benefit, or futility criteria.

A web-based system of data access was created (https://predidep.es/participantes/), from where authorized investigators and the coordinator of the trial can download forms and datasets. For privacy and security, an ID and password are required to access the data and the forms.

Quality control reports are generated for key aspects of the trial, e.g., digit preference and variability. We perform checks on missing and/or inconsistencies of the data. Following data entry, cross-form edit checks are done. Audits are carried out periodically to detect unresolved problems. Standardized edit reports summarizing database problems reassures the quality of the data.

## Discussion

Recent studies have assessed the role of the Mediterranean diet in patients with depressive disorders. Jacka et al. reported improvements in depressive symptoms in a sample of 56 depressive patients after 12 weeks of a dietary intervention with a Mediterranean diet in comparison to a social intervention [13]. Similarly, Parletta et al. observed reduced depression and higher quality of life among participants with higher MedDiet scorings in a clinical trial of 95 depressive patients based on the intervention with a Mediterranean diet supplemented with fish [14]. However, these small trials did not evaluate the long-term effect on depression of the Mediterranean diet. Moreover, the aim of the trials was to assess the role of the Mediterranean diet in the treatment of depression and not on its prevention.

The MooDFOOD prevention trial examines the feasibility and effectiveness of two different nutritional strategies (multi-nutrient supplementation and food-related behavioral change therapy). However, it does not examine the effect of a Mediterranean diet intervention in preventing depression in subjects with overweight and have elevated depressive symptoms, but with no current -or in the last six months- criteria for an episode of major depressive disorder [32].

Therefore, to the best of our knowledge, the PREDI-DEP clinical trial is the first randomized clinical trial designed to assess the role of a MDP in the prevention of recurrent depression. Preventing recurrence of depression is one of the priorities in the management of patients with depression, particularly when the number of relapses is high. Whereas the risk of recurrence is approximately 40–60% in patients with a unique depression episode, this risk raises to 90% in patients with three or more previous episodes [33, 34]. Our study provides essential evidence regarding the effectiveness and cost-effectiveness of improving the diet based on a Mediterranean eating pattern for preventing the recurrence of depression and improving the quality of life of patients with previous episodes of depression.

## Additional file

Additional file 1: 14-Item Mediterranean Diet Adherence Screener (MEDAS). Description of the MEDAS questionnaire. (DOCX 14 kb)

## Abbreviations

CIs: Confidence Intervals
DALYs: Disability-Adjusted Life Years
DMC: Data Monitoring Committee
DSM-5: Diagnostic and Statistical Manual of Mental Disorders, Fifth Edition
FFQ: Food Frequency Questionnaire
GEE: Generalized Estimating Equation
HRs: Hazard Ratios
MEDAS: Mediterranean Diet Assessment Screener
PREDIMED: *Prevención con Dieta Mediterránea* (Prevention with the Mediterranean Diet)
SCID-I: Structured Clinical Interview for DSM-IV Axis I Disorders
SF-36: Short Form (36) Health Survey
SUN: *Seguimiento Universidad de Navarra* (University of Navarra Follow-up Project)
YLDs: Years Lived with Disability
