# Supplementary material for: Preventing the recurrence of depression with a Mediterranean diet supplemented with extra-virgin olive oil. The PREDI-DEP trial: study protocol
Source: BMC Psychiatry. 2019 Feb 11;19:63. doi: 10.1186/s12888-019-2036-4 (PMC6371613; doi:10.1186/s12888-019-2036-4)
Supplement: Supplementary file 1 — 14-Item Mediterranean Diet Adherence Screener (MEDAS). Description of the MEDAS questionnaire. (DOCX 14 kb) [file 12888_2019_2036_MOESM1_ESM.docx]

**Additional file 1: 14-Item Mediterranean Diet Adherence Screener (MEDAS)**

| 1. | Use of olive oil as the main culinary fat |
| --- | --- |
| 2. | Consumption of ≥4 tablespoons/d of olive oil (including oil used for frying, salads, out-of-house meals, etc.) |
| 3. | Consumption of ≥2 servings/d of vegetables |
| 4. | Consumption of ≥3 servings/d of fruits |
| 5. | Consumption of <1 serving/d of red meat, hamburger or meat products (ham, sausage, etc.) |
| 6. | Consumption of <1 serving/d of butter, margarine, or cream |
| 7. | Consumption of <1 serving/d of sweetened and/or carbonated beverages |
| 8. | Consumption of ≥1 serving/d of wine |
| 9. | Consumption of ≥3 servings/week of legumes |
| 10. | Consumption of ≥3 servings/week of fish or shellfish |
| 11. | Consumption of <3 servings/week of commercial sweets or pastries (not homemade), such as cakes, cookies, biscuits or custard |
| 12. | Consumption of ≥3 servings/week of nuts (including peanuts) |
| 13. | Preferential consumption of chicken, turkey or rabbit meat instead of veal, pork, hamburger or sausage |
| 14. | Consumption of ≥2 servings/week of sofrito, a sauce made with tomato and onion, leek or garlic and simmered with olive oil. |
